# Niacin in the Central Nervous System: An Update of Biological Aspects and Clinical Applications

**DOI:** 10.3390/ijms20040974

**Published:** 2019-02-23

**Authors:** Valeria Gasperi, Matteo Sibilano, Isabella Savini, Maria Valeria Catani

**Affiliations:** Department of Experimental Medicine, Tor Vergata University of Rome, Via Montpellier 1, 00133 Rome, Italy; matteosibilano@libero.it (M.S.); savini@uniroma2.it (I.S.)

**Keywords:** central nervous system, diet, NAD(P), neurodegenerative diseases, niacin, nicotinamide, nicotinic acid, vitamin B_3_

## Abstract

Niacin (also known as “vitamin B_3_” or “vitamin PP”) includes two vitamers (nicotinic acid and nicotinamide) giving rise to the coenzymatic forms nicotinamide adenine dinucleotide (NAD) and nicotinamide adenine dinucleotide phosphate (NADP). The two coenzymes are required for oxidative reactions crucial for energy production, but they are also substrates for enzymes involved in non-redox signaling pathways, thus regulating biological functions, including gene expression, cell cycle progression, DNA repair and cell death. In the central nervous system, vitamin B_3_ has long been recognized as a key mediator of neuronal development and survival. Here, we will overview available literature data on the neuroprotective role of niacin and its derivatives, especially focusing especially on its involvement in neurodegenerative diseases (Alzheimer’s, Parkinson’s, and Huntington’s diseases), as well as in other neuropathological conditions (ischemic and traumatic injuries, headache and psychiatric disorders).

## 1. Introduction

**Figure 1:**
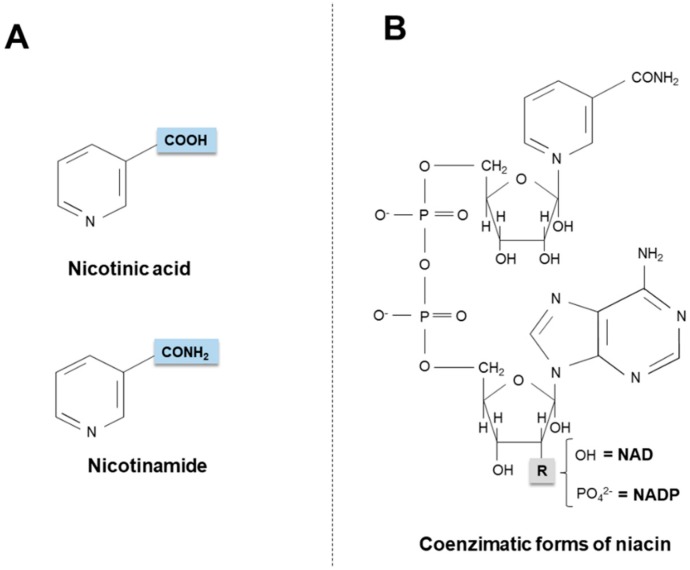
Chemical structures of niacin vitamers (**A**) and active coenzymatic forms (**B**). NAD: nicotinamide adenin dinucleotide. NADP: nicotinamide adenin dinucleotide phosphate.

Niacin (also known as “vitamin B_3_” or “vitamin PP”) is the generic descriptor for two vitamers, nicotinic acid (pyridine-3-carboxylic acid) and nicotinamide (nicotinic acid amide), that give rise to the biologically active coenzymes, nicotinamide adenine dinucleotide (NAD) and its phosphate analog, the nicotinamide adenine dinucleotide phosphate (NADP) [1] (Figure 1). The two coenzymes take part in redox reactions crucial for energy production: in particular, the pyridinic ring can accept and donate a hydride ion (:H^−^, the equivalent of a proton and two electrons), thus acting as an electron carrier. Nonetheless, NAD and NADP play different metabolic roles in the cytosol: the NADH/NAD^+^ ratio is small (about 8 × 10^−4^), thus favoring oxidative catabolism, whereas the NADPH/NADP^+^ ratio is higher (about 75), thus providing a strongly reducing environment for biosynthetic reactions [2,3].

Maintenance of the intracellular NAD pool is not only important to fuel redox metabolism, but also to support NAD-dependent, non-redox signaling pathways. NAD is indeed a substrate of ADP-ribosyltransferases that catalyze ADP-ribose transfer reactions, thus breaking down NAD to nicotinamide and ADP-ribosyl products, which play a key role in cellular signaling cascades regulating gene expression, cell cycle progression, insulin secretion, DNA repair, apoptosis and aging [4,5,6]. Finally, NAD has also been recognized as an endogenous agonist of purinergic P2Y1 and P2Y11 membrane subtype receptors, through which it inhibits neurotransmission in visceral smooth muscles [7] and activates immune cells [8,9], respectively.

## 2. Niacin Sources

Humans obtain niacin from both endogenous and exogenous sources. Only 2% of dietary tryptophan (Trp) is converted into niacin via a multistep pathway (see in next sections), occurring mainly in the liver [10]. Diet provides the vitamin as nicotinic acid, nicotinamide and Trp, as well as the active coenzymatic forms of niacin.

### 2.1. Exogenous Sources

Niacin is found in animal and vegetable foods. In meat and fish, the vitamin is present as NAD(P), whose amounts are higher in unprepared foods compared to processed foods (enzymatic hydrolysis of the coenzymes can occur during food preparation).

In mature cereal grains (particularly in corn), niacin is largely present as niacin-glycoside and, in a minor proportion, peptide-bound niacin, compounds collectively termed “niacinogens” [11]. When complexed in niacinogens, niacin is poorly available (only ~ 30%), as intestinal enzymes are not able to free niacin; nonetheless, alkali treatment of the grain increases niacin bioavailability [11]. 

Once ingested, free niacin can be adsorbed in the stomach, although the small intestine absorbs it faster. The mechanism of transport across the enterocyte brush border membrane is not fully clarified yet. Several transporters, indeed, appear to be involved in intestinal niacin uptake; among them, the most common are the human organic anion transporter-10 (hOAT-10, a proton-driven carrier that also mediates the transport of urate and *p*-aminohippurate) [12], responsible for niacin uptake at physiological concentrations [13], and the sodium-coupled monocarboxylate transporter (SMCT1 or SLC5A8, a transporter for lactate, pyruvate and short-chain fatty acids), specifically active at high pharmacological doses of nicotinic acid [14,15].

NAD and NADP are quickly hydrolyzed, by intestinal mucosa and liver glycohydrolases, to nicotinamide that is subsequently transported to tissues, where it is converted into coenzymatic forms as necessary. It seems noteworthy that nicotinamide moves freely into or out of the brain [16] and, as discussed in the next sections, such a property has important neurobiological implications.

### 2.2. Endogenous Synthesis

**Figure 2:**
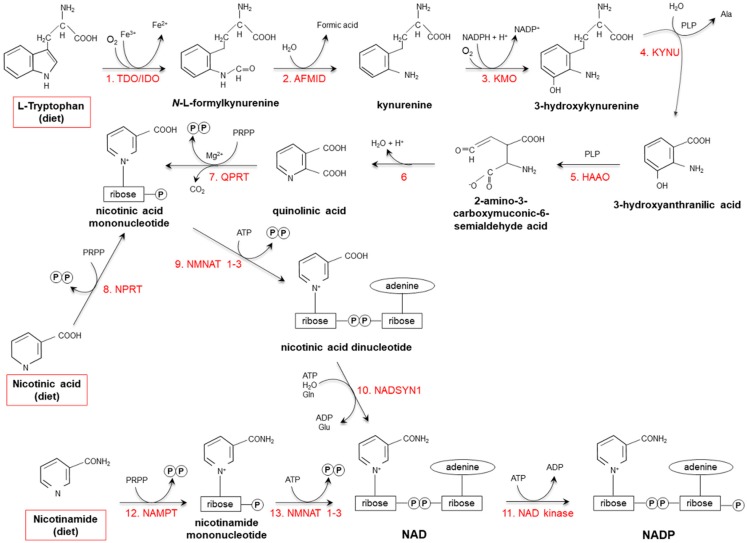
De novo synthesis of NAD(P) from tryptophan, nicotinamide and nicotinic acid. (**1**) Two iron porphyrin metalloproteins, tryptophan 2,3 dioxygenase (TDO, in the liver) and indolamine-pyrrole 2-3 dioxygenase (IDO, in extrahepatic tissues), oxidize the pyrrole moiety of Tryptophan (Trp), thus forming *N*-L-formylkynurenine. (**2**) Arylformamidase (AFMID) hydrolytically removes the formyl group producing kynurenine and is then (**3**) hydroxylated to 3-hydroxykynurenine by kynurenine-3 monooxygenase (KMO), a mitochondrial flavo-enzyme that uses O_2_ as a substrate and NADPH as a cofactor. The action of (**4**) kynureninase B (KYNU, a vitamin B_6_-dependent enzyme) and (**5**) 3-hydroxyanthranilic dioxygenase (HAAO, a nonheme iron-dependent dioxygenase) leads to production of 2-amino-3-carboxymuconic-6-semialdehyde acid, an unstable product that (**6**) spontaneously condensates and rearranges to form quinolinic acid; then, (**7**) quinolinic acid is decarboxylated and converted to nicotinic acid mononucleotide by quinolinic acid phosphoribosyltransferase (QPRT). Nicotinic acid mononucleotide is also produced through the “salvage pathway”, via the action of (**8**) nicotinic acid phosphoribosyltransferase (NPRT). The subsequent action of (**9**) nicotinamide/nicotinic acid-mononucleotide-adenylyltransferases (NMNAT1-3) and (**10**) NAD synthetase (NADSYN1) leads to the generation of NAD, which is then (**11**) phosphorylated to produce NADP. NAD can also derive directly from nicotinamide through the action of (**12**) nicotinamide phosphoribosyltransferase (NAMPT) and (**13**) nicotinamide/nicotinic acid-mononucleotide-adenylyltransferase (NMNAT1-3). Red frames: dietary precursors of NAD(P). Ala: alanine; Gln: glutamine; Glu: glutamate; PLP pyridoxal phosphate; PRPP: 5-phosphoribosyl-1- pyrophosphate.

Starting from dietary Trp, niacin is synthesized via the kynurenine pathway (KP) (Figure 2), occurring mainly in the liver and, to a lesser extent, in extrahepatic tissues (especially upon immune cell activation) [17,18,19]. 

Tryptophan 2,3 dioxygenase (TDO), catalyzing the first reaction, is the rate-limiting enzyme. Several nutritional, hormonal and physio-pathological factors affect the efficiency of this anabolic pathway. Deficiencies of vitamin B_6_, riboflavin, iron and heme (all essential cofactors for specific enzymes), as well as of vitamin B_1_ and Trp itself, slow the reaction rate [18,20]. Overall: (i) a protein-enriched diet (particularly, consumption of foods with high concentrations of leucine, such as maize or sorghum) decreases niacin biosynthesis; (ii) unsaturated fatty acid-enriched diet increases it, while saturated fatty acids do not exert any effect; (iii) the transformation ratio is higher in diets containing starch with respect to sucrose-rich diets; (iv) caloric restriction drastically suppresses biosynthesis [18,21,22,23,24,25,26].

Among hormones, estrogens, glucorticoids and thyroxine are the best characterized modulators of the KP. Estrogens enhance TDO activity; enzyme activity is triplicated in women who are pregnant or are taking oral contraceptives [27,28]. Glucocorticoids stimulate de novo synthesis, by inducing TDO via a mechanism potentiated by glucagon and inhibited by insulin and adrenaline [18,29,30]. The effects of thyroxine on TDO activity are still controversial, as some studies suggested a positive action, while others did not observe any effect [31,32,33,34]. 

Due to individual differences, it has been estimated that, in human healthy individuals, Trp is converted to niacin with an average conversion efficiency of 60:1 [35]. Therefore, niacin intakes are expressed as niacin equivalents (NE; 1 mg NE = 1 mg niacin or 60 mg Trp): Recommended Dietary Allowance for adults is 16 mg NE/day for men and 14 mg NE/day for women, with a Tolerable Upper Intake Level of 35 mg/day, based on flushing as the critical adverse effect [36].

## 3. Vitamin Catabolism

**Figure 3:**
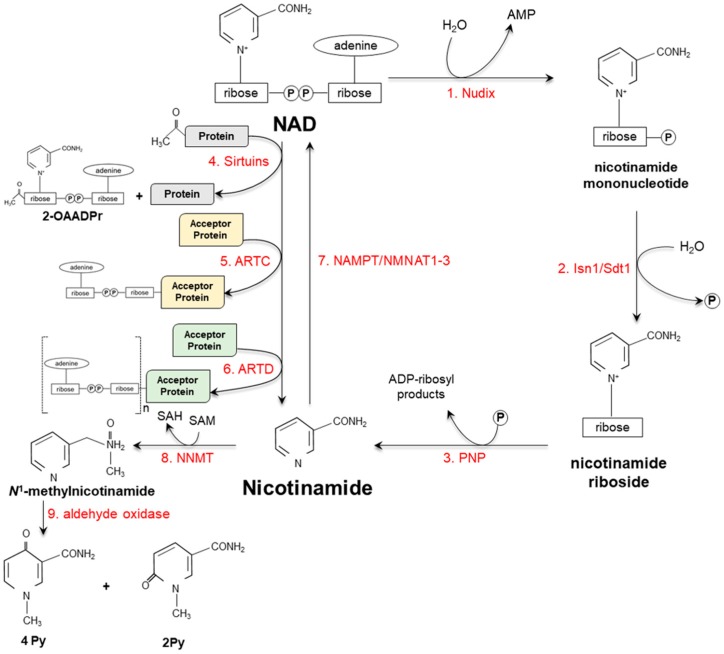
Schematic representation of distinct catabolic pathways. (**1**) NAD is hydrolyzed onto nicotinamide mononucleotide via the action of specific pyrophosphatases belonging to Nudix (nucleoside diphosphate linked to moiety X) family. (**2**) Nicotinamide mononucleotide is then dephosphorylated by Isn1 and Sdt1 cytosolic nucleotidases, which release the corresponding riboside cleaved to nicotinamide by a purine nucleoside phosphorylase (PNP) (**3**). Alternatively, NAD becomes a substrate of sirtuins (**4**), ADP-ribosyltransferases (ARTC) (**5**) and diphtheria toxin-like ADP-ribosyltransferases (ARTD) (**6**). Nicotinamide can be either re-converted to NAD by specific enzymes (**7**) (see also Figure 2) or methylated by nicotinamide-*N*-methyl transferase (NNMT) to *N*^1^-methylnicotinamide (**8**) that, in turn, (**9**) is oxidized to *N*^1^-methyl-4-pyridone-3-carboxamide (4-Py) and *N*^1^-methyl-2-pyridone-5-carboxamide (2-Py) by aldehyde oxidases. 2-OAADPr: O-acetyl-ADP ribose; NAMPT: nicotinamide phosphoribosyltransferase; NMNAT: nicotinamide/nicotinic acid-mononucleotide-adenylyltransferase; SAH: *S*-adenosylhomocysteine; SAM: *S*-adenosyl-methionine.

The tight intracellular regulation of NAD is guaranteed not only at biosynthetic but also at catabolic level; in the latter case, NAD can be either recycled or metabolized and eliminated via urine (Figure 3) [37,38,39]. 

In the recycling pathways, NAD is metabolized to nicotinamide through the action of different ADP-ribosyltransferases. Sirtuins (SIRT) are NAD-dependent deacetylases and mono-ADP-ribosyl transferases belonging to the highly conserved family of silent information regulator-2 like proteins [40,41,42]. During deacetylation, NAD is hydrolyzed and the ε-acetyl lysine residues of the target protein is transferred onto the ADP-ribose moiety, thus forming *O*-acetyl-ADP ribose (Figure 3), which is a ligand of calcium channels in the plasma membrane [43]. SIRTs deacetylate a broad spectrum of proteins, thus modulating their activity, stability or localization. Depending on the targeted protein, these enzymes affect several biological processes, including transcription, cell cycle progression, genome stability, cell death and mitochondrial biogenesis [42,44,45]. ADP-ribosyltransferases (ARTC) and diphtheria toxin-like ADP-ribosyltransferases (ARTD) catalyze mono- and poly-ADP-ribosylation, respectively, of specific amino acids (arginine, cysteine, asparagine, histidine) of membrane proteins (Figure 3), thus regulating innate immunity and cell-to-cell cross-talk, as well as cell cycle, cell death and energy metabolism [46,47,48].

Finally, NAD(P) can be hydrolyzed to nicotinamide by two ADP-ribose cyclases, namely CD38 and CD157, which also release cyclic ADP-ribose, an endogenous activator of ryanodine receptor-mediated calcium release [49,50,51,52] and suggested to be involved in pathological diseases such as cancer, neurodegeneration and autoimmune diseases [53,54,55,56].

If not recycled, nicotinamide is methylated, by the cytosolic nicotinamide *N*-methyltransferase (NNMT) that uses *S*-adenosyl-methionine (SAM) as a methyl donor, and eliminated as oxidized metabolites (Figure 3). Altered enzyme activity has been linked to several pathological conditions, including neurodegenerative diseases, obesity, type 2 diabetes and cancer [57,58,59,60,61,62,63,64]. It should be recalled that, beside nicotinamide by-products, also those deriving from conjugation of nicotinic acid to glycine (nicotinuric acid) or from its methylation (1-methylnicotinic acid) can be found in urine [65,66,67].

Due to the multiplicity of NAD-dependent biological events, which lead to NAD degradation, cells need to replenish their intracellular NAD(P) pools; inhibition of NAD biosynthesis, for example, decreases intracellular NAD content within a few hours [68].

## 4. Severe Vitamin Deficiency

Severe niacin and/or Trp deficiency leads to a variety of clinical symptoms, including diarrhea, dermatitis and dementia, collectively known as “pellagra” or “the three D disease” [69]; although this disease has become rare in developed countries, it remains endemic in underdeveloped countries [70]. Pellagra is common in people who mostly eat maize [71], as well as in malnourished and alcoholic men [26]; other risk factors leading to vitamin B_3_ deficiency are nervous anorexia [72], AIDS [73], cancer [74] and chemotherapy [75], as well as malabsorptive disorders, such as Crohn’s disease [76].

Light sensitivity is high: dermatitis derives from deficits in poly(ADP-ribose) polymerase activity that leads to impaired DNA repair. Patients can show psychiatric symptoms (i.e., depression, paranoid behaviors, suicide and aggressive tendencies) that disappear when they take niacin [77,78]; some of these symptoms are also related to deficit of serotonin that derives from Trp [78].

## 5. Pharmacological Effects of Niacin

When supplemented at physiological amounts, nicotinic acid (15–20 mg/day) and nicotinammide (300 mg/day) are effective in treating traditional pellagra [77,78]; nonetheless, at higher concentrations, they display separate additional pharmacological activities, ranging from anti-dyslipidemic to anti-inflammatory action. The first evidence of lipid-altering effects of niacin dates back to 1955, when Altschul and co-workers reported the ability of 3000 mg/day nicotinic acid (but not nicotinamide) to reduce serum cholesterol in humans [79]. An every growing body of experimental data points to beneficial effects of nicotinic acid as an anti-hyperlipidemic agent. It is now well established that nicotinic acid efficaciously: (i) inhibits free fatty acid mobilization and lipolysis; (ii) reduces hepatic triglyceride synthesis and very low density lipoprotein (VLDL) secretion; (iii) inhibits VLDL conversion into low density lipoprotein (LDL); (iv) increases serum high-density-lipoprotein (HDL) levels; (v) triggers LDL conversion from small, dense particles to large, low density particles, (vi) reduces serum lipoprotein concentrations; and (vii) increases apolipoprotein A1 [80,81]. 

To date, the underlying mechanisms are still speculative; in particular, nicotinic acid (at levels higher than those achieved with diet) has been reported to bind to and activate GPR109A and GPR109B, two G_0_/G_i_-coupled membrane receptors highly expressed in adipose tissue; nonetheless, these receptors are absent, or present only at low levels, in the liver [82]. Therefore, it is conceivable that nicotinic acid might exert its lowering-lipid effects through receptor-independent and -dependent mechanisms. 

Due to the above mentioned positive effects, in 2008, nicotinic acid was commercially available as Trevaclyn^®^, Tredaptive^®^ or Pelzont^®^, at the dose of 1.0 g (in combination with laropipram, an anti-flushing agent); this prescription product has been used to treat mixed dyslipidemic and/or primary hypercholesterolemic adults receiving statins [83]. However, results from the Atherothrombosis Intervention in Metabolic Syndrome with Low HDL/High Triglycerides: Impact on Global Health Outcomes (AIM-HIGH) trial [84], together with the Heart Protection Study 2-Treatment of HDL to Reduce the Incidence of Vascular Events (HPS2-THRIVE) trial [85,86], reported no clinical benefits (i.e., reduced risk of heart attack and stroke) from the long-lasting usage of niacin. A lack of efficacy, together with the onset of recurrent serious side effects (gastrointestinal, musculoskeletal, and skin-related), has led to drug withdrawal from the EU market.

In vitro and in vivo studies have also demonstrated that nicotinic acid (or activation of its molecular targets) exerts significant anti-inflammatory, anti-oxidant and anti-apoptotic activities in a variety of cells and tissues [87], thus being potentially beneficial for the management of several pathological conditions, including type-2 diabetes [88,89], obesity [90,91], atherosclerosis [92], kidney and lung injury [93,94,95], and hyperalgesia [96]. 

Also nicotinamide at high doses can exert specific pharmacological activities, particularly those related to cancer management. Indeed, several experimental and clinical studies have shown the ability of nicotinamide to sensitize tumors to radiation or chemotherapy [97,98,99,100]. Such an activity depends on activation of poly(ADP-ribose)-dependent apoptosis cascade, as well as on inhibition of myosin light chain kinase that, in turn, enhances microvascular flow, thus improving drug delivery and tumor oxygenation [97,98,99,100].

## 6. Niacin in the Central Nervous System

Besides dermatitis and diarrhea, niacin/tryptophan deficit symptoms also include several nervous system pathologies, such as dementia and depression, as well as other symptoms resembling those observed in neurodegenerative diseases. This evidence, together with accumulating in vitro and in vivo studies, has underlined the importance of niacin (particularly of nicotinamide) in growth and maintenance of the central nervous system (CNS) [101,102]. 

Nicotinamide biosynthesis actively occurs in the mammalian brain, which contains nanomolar-low micromolar concentrations of nicotinamide precursors derived from the KP [103,104,105]. Among them, quinolinic acid (unevenly present in different brain regions and, unlike nicotinamide, unable to cross the blood-brain barrier) displays evident neuroactivity [106]: it acts as a *N*-methyl-d-aspartate (NMDA) receptor agonist, thus causing excitotoxic neuronal lesions and oxidative stress [107]. In addition, quinolinic acid concentrations in the brain (particularly in the cortex) positively correlate with age, thus contributing to neuron synapsis dropout occurring during aging [108]. Finally, neuroinflammation, neurodegeneration and mood disturbs are accompanied by increased quinolinic acid levels in plasma and/or cerebrospinal fluid [10,109,110]. 

Among KP enzymes, TDO activity is rather low in a healthy human healthy brain [111], where it controls neurogenesis with implications in pre- and post-natal development, as well as in anxiety-related behavior [112]. TDO activity is enhanced under pathological conditions: high activity, indeed, has been found in neurodegenerative diseases and during tumor progression [113,114]. Also indolamine-pyrrole 2-3 dioxygenase (IDO) is expressed in the brain and its activity is increased upon pathological conditions, especially in depression, aging and neuroinflammatory diseases [115,116,117]. 

Like other vitamins (ascorbic acid, calcitriol and retinoic acid) [118,119,120,121,122], nicotinamide affects neurogenesis by accelerating differentiation of embryonic stem cells or neural progenitors into post-mitotic neurons [123,124]. In vitro vitamin supplementation promotes progression of undifferentiated stem cells to neural progenitors, which further mature into efficient GABAergic neurons; the pro-inducing action is time-dependent as the effects are more pronounced when the vitamin is early received early (day 0) [124]. Accordingly, decreased activity of NNMT (and, therefore, low levels of its metabolic product, *N*^1^-methylnicotinamide) is required for regulating pluripotency in stem cells: accumulation of NNMT’s substrates SAM and nicotinamide, indeed, promotes naïve to primed stem cell transition, by making SAM available for histone methylation and regulation of epigenetic events that control the metabolic changes occurring in early human development [125].

Beside the pro-differentiating action, nicotinamide also promotes neuronal survival, especially during oxidative stress conditions, and this effect is achieved via multiple mechanisms, including: (i) prevention of cytochrome c release and caspase 3- and 9-like activities, (ii) inhibition of caspase-3-mediated degradation of forkhead transcription factor (FOXO3a) and (iii) maintenance of protein kinase B (Akt)-dependent phosphorylation of FOXO3a [126]. 

CNS vascular integrity also positively correlates with NAD levels in brain, where a fine-tuned control of its metabolism occurs. As an example, heterozygous deletion of nicotinamide phosphoribosyltransferase (NAMPT) in the brain exacerbates focal ischemic stroke-induced neuronal death and brain damage [127], while its selective knock down in projection neurons of adult mice leads to motor dysfunction, neurodegeneration and death [128]. 

Finally, alterations of NAD metabolism are key features of Wallerian degeneration, a process occurring in crushed nerve fibers and leading to degeneration of the axon distal to the injury, representing an early event of age-related neurodegenerative disorders, as well as of chemotherapy-induced peripheral neuropathy [129]. By inducing intra-axonal Ca^2+^ increase through a pathway requiring the action of the pro-axon death protein SARM1, accumulation of nicotinamide mononucleotide is, indeed, responsible for loss of axonal integrity [130]. The pro-degenerative action of nicotinamide mononucleotide has also been documented during vincristine-induced degeneration in dorsal root ganglion axons [131]. Accordingly, increased activity of nicotinamide/nicotinic acid-mononucleotide-adenylyltransferase (NMNAT) 1–3 protects axons from degeneration, by either limiting nicotinamide mononucleotide levels or activating SIRT1 [132,133].

## 7. Alzheimer’s Disease

Alzheimer’s disease (AD) is a neurodegenerative disease affecting about 30 million people worldwide, whose main hallmarks are the presence of amyloid β (Aβ) plaques and neurofibrillary tangles [134].

Even if tryptophan/niacin deficiency leads to neurological symptoms that cause neurodegenerative decline [135,136,137], a cause-effect relationship between niacin and AD pathogenesis has not been established (Table 1). 

Dietary niacin may protect against AD and age-related cognitive decline, as suggested by a prospective population-based study: the Chicago Health and Aging Project (CHAP) study, considering a geographically defined community of 6158 residents aged 65 years and older, found an inverse association between AD and niacin intakes, after correction for several dietary (antioxidant nutrients, fats, folate, and vitamins B_6_, B_12_, B_1_ and B_2_) and non-dietary (age, education, race, ApoEε4) risk factors for dementia [135]. 

Although the existing epidemiologic evidence remains limited and inconclusive, niacin (especially nicotinamide) may be relevant for AD, especially keeping in mind that, by mediating key biological processes (such as energy metabolism, mitochondrial functions, calcium homeostasis, survival and cell death), NAD has lifespan-extending effects; this is particularly important in brain functions, including neurotransmission, learning and memory. NAD^+^ depletion and mitochondrial dysfunction, fundamental for synaptic plasticity, have usually been found in aging and AD onset [138,165]; accordingly, in mice models of AD, increasing NAD^+^ brain concentrations can restore mitochondrial function and antagonize cognitive decline [138,139]. Nicotinamide and/or nicotinamide mononucleotide also counteract amyloid toxicity, by reducing expression of AD-related genes (amyloid precursor protein and presenilin 1) and reactive oxygen species (ROS) generation, and by improving neuron survival: both in vitro (organotypic hippocampal slice cultures) and in vivo (AD model rats) studies have indeed underlined the protective effects of vitamin B_3_ against Aβ-induced neurotoxicity [140,141]. Moreover, the vitamin is able to lessen phosphorylated-Tau pathology in a novel AD mouse model with introduced DNA repair deficiency: nicotinamide riboside treatment significantly reduces DNA damage, neuroinflammation and cell death of hippocampal neurons, thus suggesting a therapeutic potential of NAD^+^ supplementation for AD [142]. Accordingly, the expression of *Nmnat2*, encoding for the enzyme catalyzing the conversion of nicotinamide to NAD^+^, is downregulated prior to neurodegeneration in a mouse model of dementia, and restoration of enzymatic activity has been shown to be neuroprotective against tauopathy [146]. Low levels of *Nmnat2* have also been found in AD patients and its enzymatic activity is related to clearance of tau protein [147].

Lastly, fluctuations in NAD^+^ availability can reduce AD pathology, also by modulating SIRT1 activity and slowing aging and age-associated diseases [166,167]. Several studies have underlined the key role of SIRTs in AD prevention: in particular, deacetylase activity of SIRT1 has been shown to support the non-amyloidogenic pathway of AD [143], and to counteract phenomena, like neuroinflammation, oxidative stress and mitochondrial dysfunction, contributing to, and aggravating, AD [144,145].

## 8. Parkinson’s Disease

Parkinson’s disease (PD) is a progressive disorder characterized by degeneration of dopaminergic neurons within the substantia nigra, whose main hallmarks are abnormal aggregation of the α-synuclein protein, inhibition of mitochondrial respiratory complex 1, oxidative stress and neuroinflammation. Because only 5–10% of PD cases can be ascribed to genetic predisposition, several environmental factors may play a role in sporadic forms of PD [149]. Among them, vitamin B_3_ is a promising preventive and therapeutic factor (Table 1), as it can alleviate certain types of early-onset PD symptoms. NAD^+^ levels, indeed, fall in patients with PD and, conversely, increasing niacin intake can increase dopamine synthesis in the striatum and restore optimal NAD^+^/NADH ratio needed for the activity of mitochondrial complex 1 [148]. High niacin levels can also sequester transition metal ions (including iron) that usually accumulate together with the occurrence of aggregated misfolded proteins [149,150]. Furthermore, optimal levels of vitamin B_3_ are needed for reducing oxidative stress and neuroinflammation, also implicated in PD pathogenesis: low doses of niacin alter macrophage polarization from M1 (pro-inflammatory) to M2 (anti-inflammatory) phenothype, while exogenous NADPH suppresses oxidative stress and glia-mediated neuroinflammation [151,152].

Neurons are the only cells of the brain expressing NNMT that seems to play an important role in sustaining neuron homeostasis [153]. Despite numerous investigations, the exact cause-effect relationship between NNMT and PD neuropathogenesis remains unclear. Some authors refer to NNMT as a risk factor for PD, since its levels are elevated in the cerebrospinal fluid and midbrain dopamine neurons of PD patients [153,154]. High NNMT activity is associated with low activity of mitochondrial complex 1, thus providing a link with mitochondrial dysfunction triggering neurodegeneration [154,155]. It is noteworthy that *N*^1^-methylnicotinamide (the metabolite generated by the action of NNMT) is structurally similar to *N*-methy-l-4-phenylpyridinium (MPP^+^), a toxin damaging dopamine neurons [168]. Conversely, other studies have demonstrated that the enzyme is able to (i) counteract the MPP^+^-mediated toxicity on mitochondrial complex 1, (ii) activate neuronal autophagy for balancing energy sources and cell homeostasis, and (iii) modulate neuron morphology and differentiation, by inducing neurite branching, synaptophysin expression and dopamine accumulation and release [156]. Likewise, NAD supplementation or inactivation of NAD-consuming enzymes [like PARP-1, a poly(ADP-ribose) polymerase involved in DNA repair] rescue mitochondrial defects and protect neurons against degeneration, in familial forms of PD characterized by mutations in the *pink1* gene; this finding suggests that neurotoxicity associated with mitochondrial defects may be prevented by modulating NAD^+^ salvage metabolism in order to enhance NAD availability [169].

## 9. Huntington’s Disease

Huntington’s disease (HD) is an autosomal dominant neurodegenerative disease characterized by typical progressive motor disturbances (involuntary movements of face and body, abnormalities in gait, posture and balance), psychiatric disorders (dementia) and other cognitive impairments [170]. HD is caused by a CAG expansion in the gene encoding for huntingtin (htt), located on chromosome 4; normally, the *htt* gene contains up to 35 CAG repeats, while in HD it has more than 36 CAG repeats that produce a mutant protein, with an abnormally long polyglutamine repeat (polyQ), responsible for the selective striatal degeneration of medium-sized spiny neurons and cerebral cortex [170]. In neurons, mutant htt protein aggregates, thus critically damaging cellular integrity by impairing proteostasis network, mitochondrial function and energy balance, transcriptional regulation, synaptic function and axonal transport [171].

From metabolomic studies, it has emerged that the metabolite (e.g., Trp, kynurenine, quinolinic acid and 3-hydroxykynurenine) content and activity of KP enzymes are pathologically altered in experimental HD models and human patients [109,110]. Moreover, in a *Drosophila* model of HD, disease progression has been found to be associated with a reduction in NAD levels, suggesting that dietetic or pharmacological supplementation of niacin (or its derivatives) may be useful in HD patients [157]. Several studies, indeed, have put forward a beneficial effect of vitamin B_3_ in HD (Table 1): for example, nicotinamide is protective against toxicity of polyQ proteins in *Drosophila* HD models [158], while, in transgenic mouse models, it restores brain-derived neurotrophic factor (BDNF) protein levels, increases acetylated peroxisome proliferator-activated receptor gamma coactivator 1α (PGC-1α), a master regulator of mitochondrial biogenesis, and improves motor deficits [159]. Nicotinamide effects do not depend on inhibition of mutant htt aggregation, but rather on replenishment of NAD levels required to restore energy balance dysregulation occurring in HD. 

Further insights into the neuroprotective action of nicotinamide derive from a recent study showing how nicotinamide dose-dependently prevents motor abnormality in 3˗nitropropionic acid-induced rat model of HD. Such an effect seems to be linked to prevention of oxidative stress (i.e., decrease in malondialdehyde and nitrites, increase in glutathione), as well as to inhibition of neuronal death in the striatum, most likely through a PARP-1-dependent mechanism [160]. Accordingly, PARP-1 is activated in response to 3- nitropropionic acid-induced neurotoxicity [161] and PARP-1-triggered astrocyte death is prevented by nicotinamide [162]. Like PARP-1, SIRTs are involved in HD neurodegeneration. In particular, SIRT1 is impaired, most likely because of the ability of mutant htt to directly bind and inhibit it; subsequent hyperacetylation and inactivation of specific genes lead to abrogation of the deacetylase pro-survival action [172]. Accordingly, increased SIRT1 activity rescues neurons from mutant htt toxicity and ameliorates pathological mechanisms underlying HD onset [163,164].

All these findings are somewhat controversial, since other studies reported opposite effects. By using the YAC128 transgenic model (expressing the full-length human mutant *htt* gene), Naia and co-workers [173] compared the effects of nicotinamide (a SIRT1 inhibitor) and resveratrol (a SIRT1 activator), both in vitro and in vivo. Both compounds were able to modify histone H3 acetylation and counteract mitochondrial dysfunction in striatal and cortical neurons isolated from YAC128 embryos; nonetheless, only resveratrol ameliorated energy homeostasis and mitochondrial function, as well as motor coordination, in in vivo HD models. Counterintuitively, in vivo nicotinamide supplementation (especially at high concentrations) did not cause any improvement in motor behavior and, furthermore, it worsened motor performance in wild-type control mice. The harmful action has further been documented in other neurodegenerative pathologies: in lactacystin-lesioned rats (an in vivo model of PD), one-month nicotinamide supplementation leads to SIRT1 inhibition and over-expression of neurotrophic and anti-apoptotic factors, nonetheless it exacerbated degeneration of dopaminergic neurons [174].

Therefore, these data underscore the need of full understanding the pathogenetic mechanisms of neurodegeneration, before suggesting any therapeutic challenge to slow down the progression of symptoms.

## 10. Other Neurological Diseases

Besides neurodegeneration, the impact of vitamin B_3_ on CNS has also been investigated in other neuropathological conditions, among which (i) ischemic and traumatic injuries, (ii) headache and (iii) psychiatric disorders (Table 2).

### 10.1. Ischemic and Traumatic Injuries

When brain cells are deprived of oxygen for more than a few seconds, severe damage occurs, culminating in cell death, through cerebral infarction or ischemic stroke. During reperfusion following a transient ischemic episode, other significant harm (including oxidative stress, leukocyte infiltration, mitochondrial dysfunction, platelet activation and aggregation, complement activation, and blood-brain-barrier disruption) also occur, contributing to neurological dysfunction [200]. 

Re-oxygenation of neural tissue dramatically impairs NAD^+^/NADH recycling, an event known as NADH hyperoxidation [201]. Over the years, the potential neuroprotective and neurorestorative role of vitamin B_3_ in ischemic brain injury has extensively been demonstrated in in vitro and in vivo models. By using hippocampal slices, Shetty and co-workers [183] demonstrated that NADH hyperoxidation is correlated with diminished neuronal recovery that can be rescued by enhancing NAD^+^ levels. Pre-treatment of brain tissue with nicotinamide (to enhance NAD^+^ availability) or PARP-1 antagonists (to lessen NAD^+^ consumption), indeed, prevents mitochondrial dysfunction, improves ATP content and stimulates neuronal recovery, during re-oxygenation [183]. Nicotinamide seems to be efficacious also when provided after ischemia-reperfusion injury. For example, rats receiving a single high dose or repeated low doses of vitamin B_3_ after cardiac arrest show reduced neurologic deficits, hippocampal apoptosis, axonal injury and microglial activation in corpus callosum [181]. Nicotinamide-dependent mechanisms underlying these effects include restoration of NAD(P) content and decrease in oxidative stress, along with repression of mitogen-activated protein kinase signaling and caspase 3 cleavage in brain tissue [181]. These data are in agreement with previous reports showing how nicotinamide significantly reduces brain infarct size and improves neurological deficits in different rat strains [202,203,204,205,206]. Interestingly, neurorestoring effects are also present when niacin is provided several hours after ischemic damage: when administrated 24 h after a middle cerebral artery occlusion, Niaspan (a FDA-approved prolonged release formulation of niacin) increases local cerebral blood flow, promotes angiogenesis (via angiopoietin1/Tie2, Akt and endothelial NOS pathways) and arteriogenesis (via tumor necrosis factor-alpha-converting enzyme and Notch signaling), and ameliorates functional deficits [184,185].

NAMPT is critically involved in vitamin B_3_ effects. Proof of its key role include: (i) decreased NAMPT activity significantly worsens post-ischemic brain damage [189,190]; (ii) heterozygous *Nampt* deletion aggravates brain damage following photothrombosis-induced focal ischemia [190], (iii) *Nampt* over-expression reduces infarct size [191]. Accordingly, when intraventricularly injected, the NAMPT substrate nicotinamide mononucleotide reverts the detrimental effects of FK866 (a NAMPT inhibitor) [189], ameliorates hippocampal injury and improves neurological outcome, by decreasing poly-ADP-ribosylated proteins and NAD^+^ catabolism [182].

The evidence of niacin efficacy against ischemic insult strongly prompted researchers to investigate its validity in other brain injuries, including traumatic brain injury (TBI). Rats receiving niacin following a cortical contusion injury (an experimental model of TBI) show reduced behavioral deficits and improved long-lasting functional recovery [175,176,177,178,179,180].

Regardless the type of brain injury, greater beneficial effects have been observed when vitamin B_3_ was administrated in combination with other “natural compounds”. Co-administration of nicotinamide and progesterone not only increases function recovery, reduces lesion cavitation and tissue loss in both injured cortex and reactive astrocytes, but also modulates expression of genes involved in inflammatory and immune responses, including *Ccr1* (chemokine (C-C motif) receptor 1), *Clec4e* (C-type lectin domain family 4, member 3), *Fn1* (fibronectin 1), *Hmox1* (heme-oxygenase 1), *Hspb1* (heat shock protein b1), *Igf1* and *2* (insulin like growth factor 1 and 2), *Il1β* (interleukin 1 β), *Il16* and *18* (interleukin 16 and 18), *Mmp8* and *9* (matrix metallopeptidase 8 and 9), *Niacr1* (niacin receptor 1) and *Ptgs2* (prostaglandin-endoperoxide synthase 2) [187,188]. In an in vitro model of ischemia-reperfusion injury, combination of niacin and selenium (at clinically relevant doses) synergistically attenuates cortical cell injury, by increasing Akt phosphorylation and expression of nuclear factor erythroid 2-related factor 2, stimulating glutathione redox cycle and reducing hydrogen peroxide levels [186].

### 10.2. Headache

Affecting more than fifty percent of adult population, headache represents one of the most widespread causes of disability worldwide. Pathogenic mechanisms underlying migraine and tension-type headache (the most common primary headache types) are mostly superimposable: headache, indeed, is triggered by trigeminovascular complex activation that leads to intracranial vessel vasoconstriction followed by extracranial vessel vasodilation and perivascular nociceptive nerve activation. Pressure changes in cerebrospinal fluid and/or intracranial veins are also involved [207,208].

Some nutrients, such as magnesium, carnitine, coenzyme Q10, vitamins (B_2_, B_12_, D) and alpha lipoic acid, can be used as preventive compounds able to counteract headache migraine attacks [209]. When orally, intramuscularly or intravenously administrated, vitamin B_3_ (especially, nicotinic acid) has therapeutic effects in headache management [210,211,212,213,214,215]. It has been proposed that niacin might exert beneficial effects by acting at both central and peripheral levels; in particular, it efficaciously dilates intracranial vessels and subsequently contracts extracranial vessels, favoring, in parallel, the release of compounds leading to peripheral vasodilation and cutaneous flushing. Taking into account that plasma content of serotonin inversely correlates with headache onset, niacin acts, at the central level, by increasing Trp-dependent synthesis of serotonin, via feedback inhibition of the KP [194]. At the peripheral level, pharmacological doses of nicotinic acid increase skin biosynthesis of prostaglandin D2 [195] and the plasma content of its by-product 9a,11b-PGF2, in healthy volunteers [196]. 

It should also be mentioned that alterations of mitochondrial regulatory networks play a key role in migraine pathophysiology [192,193]. Therefore, by enhancing substrate availability for complex I and reducing lactate concentration, niacin might restore mitochondrial energy metabolism and ameliorate blood flow and oxygenation in sore skeletal muscle, especially in tension-type headache.

### 10.3. Psychiatric Disorders

A large number of mental disorders have been shown to be influenced by dietary habits, leading to the development of nutritional guidelines for prevention and/or treatment of psychological disorders, including depression, anxiety, schizophrenia, bipolar disorders and psychological distress. In particular, vitamin B_3_ dysmetabolism may be linked with some of these neuropsychiatric disorders, although the literature reports conflicting data: as an example, an epidemiologic study conducted on 140 subjects (73 controls and 67 patients with schizophrenia) has revealed that affected individuals show significantly lower dietary intakes of specific nutrients, including niacin [197], whereas a 1-year case-control study performed on 101 controls and 128 cases of schizophrenia found a direct relationship between the disease and nicotinamide levels [198].

The main etiological factors involved in mood disorders appear to be metabolites produced in the KP, as a consequence of the shunt of Trp from serotonin synthesis to kynurenine formation [216]. Serotonergic neurotransmission, indeed, is compromised in the brain of depressed individuals, as a result of activated KP. Since IDO activity is induced under inflammatory and oxidative conditions, and KP is mostly active in astrocytes and microglia (also responsible for production of pro-inflammatory mediators), it has been proposed that unbalanced KP leads to impaired glial-neuronal network, thus priming the CNS against psychological stress [217]. In human postmortem studies, high levels of kynurenic acid (deriving from transamination of kynurenine instead of hydroxylation, see Figure 2) have been found in the prefrontal cortex of schizophrenic individuals; this finding may have clinical relevance, as kynurenic acid is an antagonist of both NMDA and nicotinic acetylcholine receptors, whose blockade is involved in cognitive deficits associated with the disease [218]. Like schizophrenia, alterations in kynurenine precursor have also been observed in bipolar disorder, although, in this case, nicotinamide levels represent a better prognostic factor; indeed, higher nicotinamide levels are correlated with suicide as a cause of death in bipolar patients (1.3-fold increase with respect to bipolar individuals who died from other causes) [219].

The immune-related imbalance of KP can also be responsible for dendritic atrophy and anhedonia associated with major depressive disorder (MDD): comparison between controls (20 healthy subjects) and patients (29 unmedicated individuals who met the Diagnostic and Statistical Manual of Mental Disorders-IV criteria for MDD) revealed, in the MDD group, a lower neuroprotective index [ratio between kynurenic acid (neuroprotective) and quinolinic acid (neurotoxic)], which was negatively correlated with anhedonia and positively correlated with hippocampal and amygdala volume [220]. According to these data, *tdo* knock-out mice show, if compared to wild-type littermates, higher levels of Trp and serotonin in the hippocampus and midbrain, which are connected to increased neurogenesis and amelioration of anxiety-related behavior [221].

Together, such findings suggest a potential antidepressant effect of vitamin B3 or its related products. In a patient with bipolar type II disorder, nicotinamide supplementation (1 g three times daily) for over 11 years has proven effective in maintaining the patient stable and calm [199]. Although a single case report is weak and does not allow us to generalize the results, it may aid in the understanding the potential additional mechanisms accounting for mental disorders.

## 11. Conclusions

A growing body of evidence highlights the key role of vitamin B3 in neuronal health. What is emerging is that niacin bioavailability is crucial for neuronsurvival and functions: indeed, vitamin deficiency has been recognized as a pathogenic factor for neurological deficits and dementia, as well as for neuronal injury and psychiatric disorders. 

**Figure 4:**
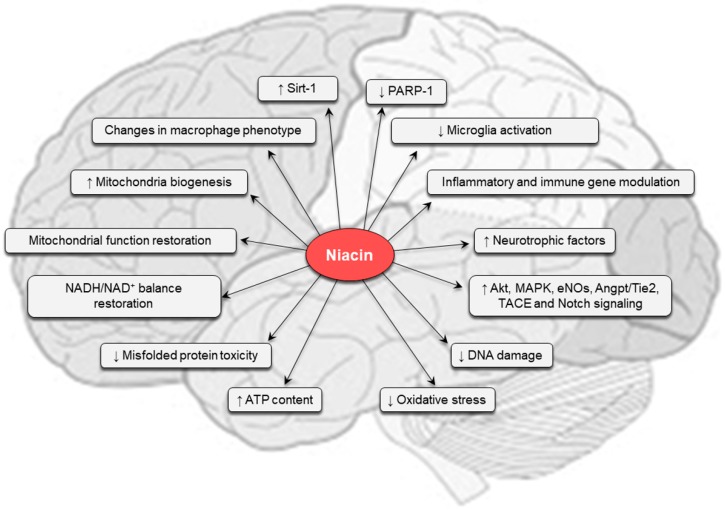
Main molecular mechanisms underlying beneficial effects of niacin in the CNS under physio-pathological conditions. See text for further details. Akt: protein kinase B; Angpt: angiopoietin1; eNOS: endothelial nitric oxide synthase; MAPK: mitogen-activated protein kinase; PARP-1: poly(ADP-ribose) polymerase-1; SIRT1: sirtuin-1; TACE: tumor necrosis factor-alpha-converting enzyme.

Several molecular mechanisms are influenced by vitamin B3 (Figure 4), often strictly linked each other, thus making it difficult to define the precise mechanisms of action of this dietary metabolite. Although further research is needed, it may be speculated that optimal dietary intake of the vitamin will support neuronal health and delay neurodegeneration.

## Figures and Tables

**Table 1:** Main findings on the role of niacin in neurodegeneration.

	Effector	Main Findings	Ref.
Alzheimer’s disease	Niacin	Inverse association between AD and dietary niacin intakes	[135]
	NAD^+^	High brain levels restore mitochondrial function and antagonize cognitive decline	[138,139]
	Nam/Nam mononucleotide	Protect against Aβ-induced neurotoxicity via reduction of *APP* and *PSEN-1* expression and ROS levels	[140,141]
	Nam riboside	Reduces DNA damage, neuroinflammation and cell death of hippocampal neurons	[142]
	SIRT1	Supports the non-amyloidogenic pathway of AD Lessens AD neuroinflammation, oxidative stress and mitochondrial dysfunction	[143] [144,145]
	NMNAT1-3	Protects against axon degeneration via reduction of nicotinamide mononucleotide levels and SIRT1 activation	[132,133]
	NMNAT2	Activity downregulated prior to neurodegeneration; restoration of activity is neuroprotective against tauopathy Low gene expression in AD patients	[146] [147]
Parkinson’s disease	Niacin	Increased intake enhances striatal dopamine synthesis and restores optimal NAD^+^/NADH ratio High levels sequester transition metal ions Low doses impact macrophage polarization from M1 (pro-inflammatory) to M2 (anti-inflammatory) profile	[148] [149,150] [151]
	NAD^+^	Decreased levels in PD patients	[148]
	NADPH	Inhibits MPTP^+^-induced oxidative stress and glia-mediated neuroinflammation	[152]
	NNMT	High levels in the cerebrospinal fluid and midbrain dopamine neurons of PD patients High activity associated with low activity of mitochondrial complex 1; it counteracts the MPP^+^-dependent toxicity on mitochondrial complex 1 and activates neuronal autophagy Induces neurite branching, synaptophysin expression and dopamine release	[153,154] [154,155] [156]
Huntington’s disease	NAD	Low levels correlate with disease progression in *Drosophila* HD model	[157]
	Nam	Protects against the toxicity of polyQ proteins in *Drosophila* HD models Restores BDNF protein levels, increases acetylated PGC-1α, improves motor deficits Prevents motor abnormality via PARP-1-dependent inhibition of neuronal death and oxidative stress	[158] [159] [160,161,162]
	SIRT1	Rescues neurons from mutant huntingtin toxicity Ameliorates pathological mechanisms underlying disease onset	[163,164]

AD: Alzheimer’s disease; APP: amyloid precursor protein; BDNF: brain-derived neurotrophic factor; HD: Huntington’s disease; MPTP^+^: *N*-methy-l-4-phenylpyridinium; Nam: nicotinamide; NMNAT: nicotinamide/nicotinic acid-mononucleotide-adenylyltransferases; NNMT: Nicotinamide *N*-Methyltransferase; PARP-1: poly(ADP-ribose) polymerase-1; PD: Parkinson’s disease; PGC-1α: peroxisome proliferator-activated receptor gamma coactivator 1α; PSEN-1: presenilin-1; ROS: reactive oxygen species; SIRT1: sirtuin1.

**Table 2:** Main findings on the role of niacin in other neurological diseases.

	Effector	Main Findings	Ref.
Ischemic and traumatic injuries	Niacin	Diminishes TBI-dependent behavioral deficits and improves functional recovery	[175,176,177,178,179,180]
	Nam	Reduces neurologic deficits, hippocampal apoptosis, axonal injury and microglial activation in corpus callosum and oxidative stress; restores NAD(P) content; represses MAPK signaling and caspase 3 cleavage	[181]
	Nam mononucleotide	Ameliorates hippocampal injury and improves neurological outcome, by decreasing poly-ADP-ribosylated proteins and NAD^+^ catabolism	[182]
	Nam/PARP-1 antagonists	Pre-treatment improves ATP content and neuronal recovery during re-oxygenation	[183]
	Niaspan (niacin)	Increases local cerebral blood flow; promotes angiogenesis via angpt/Tie2, Akt and eNOS pathways; promotes arteriogenesis via TACE and Notch signaling; ameliorates functional deficits	[184,185]
	Niacin plus selenium	Attenuate cortical cell injury, via an increase in Akt phosphorylation and expression of Nrf2; reduce oxidative stress.	[186]
	Nam plus progesterone	Increase function recovery; reduce lesion cavitation and tissue loss; modulate expression of inflammatory and immune genes	[187,188]
	NAMPT	Decreased activity exacerbates post-ischemic brain damage Heterozygous gene deletion aggravates brain damage following photothrombosis-induced focal ischemia Gene over-expression reduces infarct size	[189,190] [190] [191]
Headaches	Niacin	Restores mitochondrial energy metabolism Ameliorates blood flow and oxygenation in contracted skeletal muscle	[192,193]
	Nicotinic acid	Dilates intracranial vessels and contracts extracranial vessels; increases skin biosynthesis of prostaglandin D2; rises plasma content of 9a,11b-prostaglandin F2	[194,195,196]
Psychiatric disorders	Niacin	Low dietary intakes in neuropsychiatric patients	[197]
	Nam	Positive correlation between vitamin levels and schizophrenia Chronic supplementation effective in maintaining a bipolar type II patient stable and calm	[198] [199]

Akt: protein kinase B; Angpt: angiopoietin1; eNOS: endothelial Nitric oxide synthase; MAPK: mitogen-activated protein kinase; Nam: nicotinamide; NAMPT: nicotinamide phosphoribosyltransferase; Nrf2: Nuclear factor (erythroid-derived 2)-like 2; PARP-1: poly(ADP-ribose) polymerase-1; TACE: tumor necrosis factor-alpha-converting enzyme; TBI: traumatic brain injury.

## Abbreviations

AD	Alzheimer’s disease
Akt	protein kinase B
ARTC	ADP-ribosyltransferases
ARTD	diphtheria toxin-like ADP-ribosyltransferases
CNS	central nervous system
FOXO3a	forkhead transcription factor
HD	Huntington’s disease
HDL	high density lipoprotein
hOAT-10	human organic anion transporter-10
Htt	huntingtin
IDO	indolamine-pyrrole 2-3 dioxygenase
KP	kynurenine pathway
LDL	low density lipoprotein
MDD	major depressive disorder
MPP^+^	*N*-methy-l-4-phenylpyridinium
NAD(P)	nicotinamide adenine dinucleotide (phosphate)
NAMPT	nicotinamide phosphoribosyltransferase
NE	niacin equivalents
NMDA	*N*-methyl-D-aspartate
NNMT	*N*-methyltransferase
PARP	poly(ADP-ribose) polymerase
PD	Parkinson’s disease
polyQ	polyglutamine repeat
ROS	reactive oxygen species
SAM	*S*-adenosyl-methionine
SIRT	sirtuin
SMCT1/SLC5A8	sodium-coupled monocarboxylate transporter
TBI	traumatic brain injury
TDO	tryptophan 2,3 dioxygenase
Trp	tryptophan
VLDL	very low density lipoprotein
